# Liposome-mediated delivery of challenging chemicals to aid environmental assessment of Bioaccumulative (B) and Toxic (T) properties

**DOI:** 10.1038/s41598-020-66694-3

**Published:** 2020-06-16

**Authors:** Mafalda Castro, Dennis Lindqvist

**Affiliations:** Department of Environmental Science, Stockholm University, 106-91 Stockholm, Sweden

**Keywords:** Environmental sciences, Environmental chemistry

## Abstract

Standard aquatic toxicity tests of chemicals are often limited by the chemicals’ water solubility. Liposomes have been widely used in the pharmaceutical industry to overcome poor pharmacokinetics and biodistribution. In this work, liposomes were synthesized and used in an ecotoxicological context, as a tool to assure stable dosing of technically challenging chemicals to zooplankton. Three chemicals with distinctly different characteristics were successfully incorporated into the liposomes: Tetrabromobisphenol A (TBBPA, log *K*_ow_ 5.9, p*K*_*a1*_ 7.5, p*K*_*a2*_ 8.5), chlorinated paraffin CP-52 (log *K*_ow_ 8–12) and perfluorooctanoic acid (PFOA, p*K*_*a*_ 2.8). The size, production yield and stability over time was similar for all blank and chemical-loaded liposomes, except for when the liposomes were loaded with 10 or 100 mg g^−1^ PFOA. PFOA increased the size and decreased the production yield and stability of the liposomes. *Daphnia magna* were exposed to blank and chemical-loaded liposomes in 48 hour incubation experiments. A dose-dependent increase in body burden in *D. magna* and increased immobilization (LD_50_ = 7.6 ng CPs per individual) was observed. This confirms not only the ingestion of the liposomes but also the successful internalization of chemicals. This study shows that liposomes can be a reliable alternative to aid the study of aquatic toxicity of challenging chemicals.

## Introduction

Standard aquatic toxicity tests (e.g., OECD guidelines for the testing of chemicals) are valuable tools that help to decrease uncertainty and increase reproducibility of results. Data originated from standard aquatic toxicity tests are often used for hazard identification during environmental risk assessment of chemicals^1,2^. The most commonly used aquatic toxicity testing guidelines, both in regulatory and scientific contexts, have been published by the Organisation for Economic Co-operation and Development (OECD). These guidelines were originally developed for readily water-soluble and stable chemicals, and it has been historically difficult to accommodate these tests for the whole range of chemicals in the marketplace^3^. The most problematic physical-chemical properties while performing aquatic toxicity testing are low water solubility (high log *K*_ow_ (> 6)), high volatility (log *K*_aw_ > 1), pH sensitive speciation, and surface-active properties. Hydrophobic organic chemicals (such as polychlorinated biphenyls (PCBs), polycyclic aromatic hydrocarbons (PAHs), and chlorinated paraffins (CPs)) are traditionally difficult to test due to their low water solubilities, leading to lowered bioavailability and, therefore, uncertain exposure in water^4–6^. Passive dosing *via* silicone has been developed to accommodate this group of chemicals, allowing stable water exposures at environmentally relevant concentrations^7–9^. Aquatic toxicity tests of volatile chemicals such as siloxanes and kerosene present difficulties due to the chemicals tendency to evaporate^10,11^. Headspace passive dosing methods have been developed and validated for these type of chemicals, where the chemicals are delivered to the water via air-water equilibrium from a saturated headspace^11^. pH sensitive chemicals are problematic as the ratio between the conjugated acid- and base-form will differ with the pH. For example, the water solubility of tetrabromobisphenol A (TBBPA) varies from 0.17 mg L^−1^ at pH 3 (100% neutral form) to 30.5 mg L^−1^ at pH 8 (when double negatively charged)^12^. Lastly, amphiphilic chemicals such as per- and polyfluoroalkyl substances (PFAS) are of particular complexity due to their simultaneous hydrophobicity and lipophobicity.

Liposomes are thought to have been first discovered by the German bacteriologist Paul Ehrlich in the 1900s and described as the “ultimate magic bullet”. Thereafter, these vesicles have been extensively studied in pharmaceutical sciences as drug carriers, as vectors to effectively stabilize therapeutic chemicals, and to assure cellular and tissue uptake^13,14^. Liposomes are spherical vesicles most often composed of a phospholipid bilayer with an internal hydrophilic compartment (similar to e.g., cell vacuole). This structure allows a unique ability to trap both lipophilic and hydrophilic chemicals. Theoretically, a lipophilic drug will be trapped in the hydrophobic intermembrane space, whereas a hydrophilic (or ionic) drug will be trapped in the aqueous vacuole. The negatively charged phospholipid heads and neutral chains should trap both ionic and amphiphilic chemicals, thus making liposomes an incredibly versatile tool to encapsulate a wide-range of chemicals^15–18^. Furthermore, conventional liposomes are expected to be biologically inert particles, due to their natural phospholipidic composition, leading to null or minimal toxicity^15–18^, as opposed to traditionally used solvent carriers such as DMSO or methanol. Liposomes can be altered and characterized according to their size, charge, lipid composition and surface modifiers (with target ligands such as specific antibodies, peptides or fluorescent agents)^14^.

Liposomes have previously been used in ecological contexts mostly as nutrient carriers (polyunsaturated fatty acids) to many different marine and freshwater zooplankton species such as *Artemia nauplii*^19^*, Daphnia pulex*^20^, and *Calanus finmarchicus* and *Calanoides acutus*^21^. Buttino and co-workers developed giant liposomes (5–40 μm) with a high molecular mass fluorescent chemical (fluorescein isothiocyanate-dextran) encapsulated, and demonstrated their uptake as food in marine copepods^22^.

The hypothesis of this study was that liposomes could be used as a tool for the delivery of highly hydrophobic, ionic, and amphiphilic chemicals to *D. magna*, and thus could be established as a means to aid the aquatic toxicity testing of challenging chemicals. The hypothesis was tested by a) synthesizing blank and chemical-loaded liposomes, and thereafter determining size distribution, stability over time and chemical encapsulation yield, and b) testing the ingestion and liposome-mediated delivery of challenging chemicals to *D. magna*. We used three model chemicals: i) tetrabromobisphenol A (pH sensitive speciation), ii) perfluorooctanoic acid (amphiphilic chemical), and iii) chlorinated paraffins (highly hydrophobic chemicals).

## Results

### Physical-chemical properties of the liposomes

**Figure 1:**
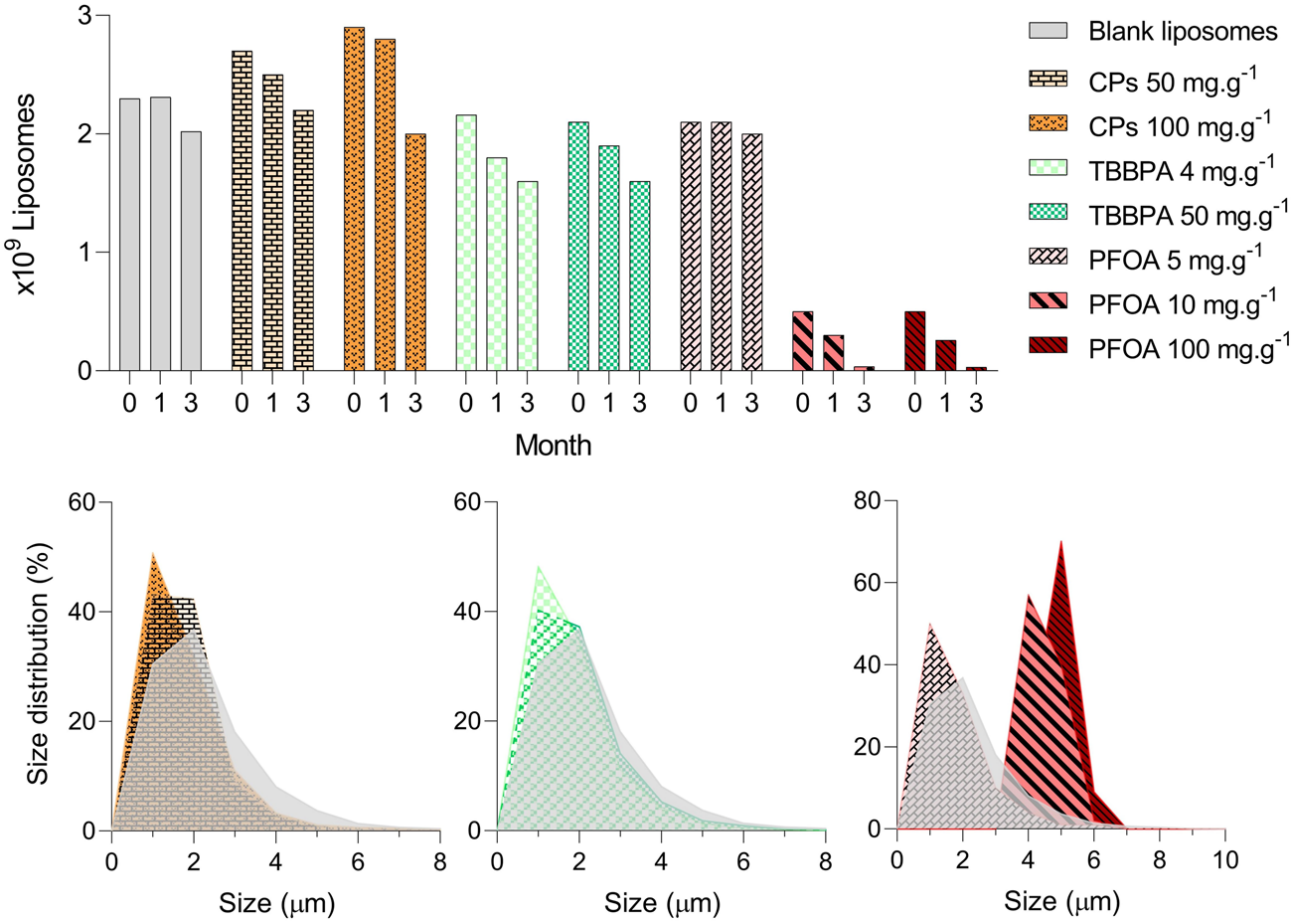
Physical characteristics of the blank and chemical-loaded liposomes. Stability over time (number of liposomes at 0, 1 and 3 months after preparation, top) of the blank and chemical-loaded liposomes. The number of liposomes per batch over time was used as a proxy for disintegration. Size distribution (%, 1 up to 10 μm diameter, bottom) of the blank and chemical loaded liposomes at different concentrations: 50 and 100 mg g^−1^ CPs (bottom left), 4 and 50 mg g^−1^ TBBPA (bottom center) and 5 and 10 and 100 mg g^−1^ PFOA (bottom right).

The production of blank liposomes was possible with relatively high precision regarding size and concentration, producing on average 2.3 × 10^9^ liposomes from a single batch (from 130 mg of material, RSD = 3%, n = 3). The production yield varied between 89 and 126% of the control when TBBPA and CPs were incorporated into the liposomes (Supplementary Table S1). However, when PFOA was incorporated at a level of 10 and 100 mg g^−1^ liposomes, the liposome yield was only 25 and 14% compared to the control respectively (Supplementary Table S1). Liposome concentration decreased slightly three months after preparation, and the 10 and 100 mg g^−1^ PFOA liposomes decreased nearly 10-fold after three months (Fig. 1, Supplementary Table S1). The median diameter of the liposomes consistently ranged between 2.0 and 2.2 µm in diameter (Supplementary Table S1) for the CP, PFOA (5 mg g^−1^) and TBBPA loaded liposomes, while the blank liposomes showed a slightly higher median diameter of 2.6 µm. Less than 10% of these liposomes were smaller than 1.3 µm in size (Supplementary Table S1). However, as can be observed in Fig. 1, liposomes loaded with higher amounts of PFOA (10 and 100 mg g^−1^) increased in size, up to 5.2 and 5.4 μm in median diameter (Supplementary Table S1). Incorporation of CPs and TBBPA in the liposomes was successful, with 90 and 84% incorporation yield of the respective chemical, at 50 mg chemical per g liposomes (Supplementary Table S2). However, incorporation was much lower for PFOA at 5 mg g^−1^, where only 30% of the added chemical was actually incorporated into the liposomes (Supplementary Table S2).

Directly following the dilution of the chemically loaded liposomes (diluted to 0.5 × 10^5^ liposomes mL^−1^) an immediate release of part of the incorporated chemical to the to the fresh M7 media was observed, see Supplementary Table S2 (*t*_≈0_). PFOA-loaded liposomes leaked the most with a release of up to 58% of the PFOA into the water phase (Supplementary Table S2), followed by TBBPA with a release of up to 27.5% into the water phase. From measurements taken after 0, 24 48, and 120 hours following dilution of the liposomes with M7 medium, it was observed that the amount of chemical in water (%) did not significantly increase over time after the initial release.

### Ingestion of liposomes by *D. magna*

At a concentration of 1 to 3 × 10^5^ particles mL^−1^, i.e. the same concentration as used, on average, for algae cells (*P. subcapitata*) to feed *D. magna*, liposomes caused a significant decrease in survival after 48 hours incubation (Supplementary Fig. S1). At 0.5 × 10^5^ liposomes mL^−1^, after 48 hours incubation, the survival was observed to be consistently between 90 and 100%, which is permissible during aquatic toxicity testing as per guideline criteria. During ingestion experiments, it was observed that *D. magna* ingested blank liposomes at the same rate as *P. subcapitata* (Supplementary Fig. S2). However, daphnids ingested significantly less of the PFOA and TBBPA-loaded liposomes (p < 0.05, Supplementary Fig. S2). The animals also ingested less CP-loaded liposomes but this was not statistically significant. The ingestion of liposomes by the animals was further confirmed with the aid of fluorescence microscopy (Supplementary Fig. S3). The fluorescence observed in the gut (arrow in Supplementary Fig. S3) and in the body indicates that the animals ingested and digested the Nile Red (NR) stained liposomes. In order to show that the fluorescence observed in the animals’ gut is a result of the NR-stained liposomes and not the NR staining the gut particles, a few animals were stained via water (15 μM NR in M7 medium, no liposomes, Supplementary Fig. S3D). A different pattern was observed where the lipid droplets in the midgut area (lipid reserves) are stained instead of the lower part of the gut.

### Liposome delivery of chemicals to *D. magna*

**Figure 2:**
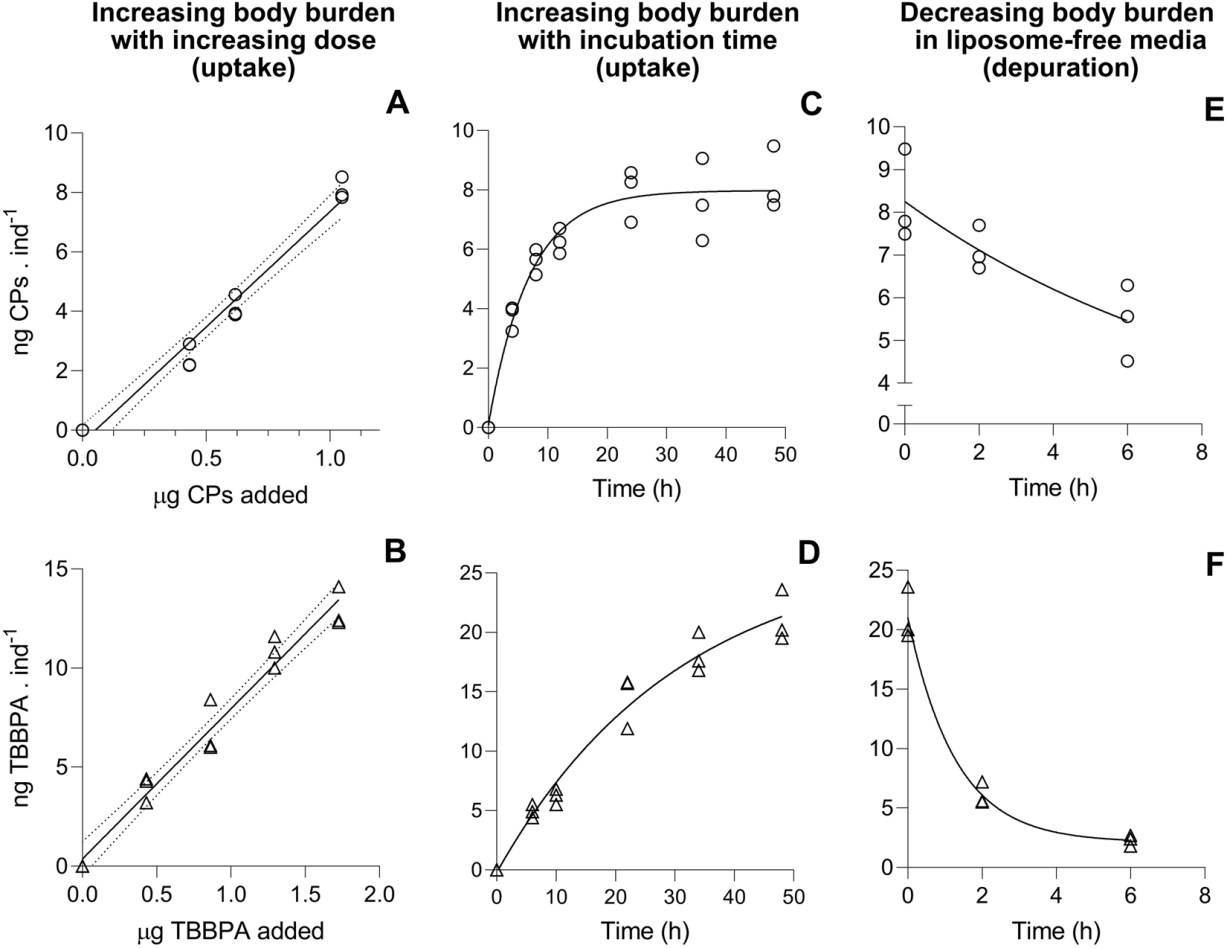
Variation of body burden (ng CPs (top) or TBBPA (bottom) per individual *D. magna*) at different doses and over time. A and B: Linear regression for the body burden as a function of the amount of chemical added (μg added) to the system via liposomes after 48 hours incubation (${ng}\,{in}{{d}}^{-1}={a}\times {\mu }{g}\,{added}+{b}$; R^2^ = 0.97 for both regressions, Supplementary Table S3) with the 95% confidence intervals of the regression dotted; C and D: increase of body burden over time (between 4 and 48 hours of incubation with chemical-loaded liposomes) at constant chemical dose (1.1 μg CPs and 2.2 μg TBBPA *via* liposomes) and constant liposome concentration of 0.5 × 10^5^ liposomes mL^−1^. Data was fitted with an exponential plateau model (${ng}\,{in}{{d}}^{-1}={Plateau}-({Plateau}-{ng}\,{in}{{d}}_{0}^{-1})\times {{e}}^{-{kt}};$ R^2^ = 0.94 for CPs, R^2^ = 0.97 for TBBPA, Supplementary Table S4); E and F: decrease of body burden in *D. magna* after being moved to liposome and chemical free M7 media, with 1 mg L^−1^ cellulose (after 48 hours incubation with 1.1 μg CPs and 2.2 μg TBBPA *via* liposomes). Data was fitted with an exponential (one-phase) decay model (${ng}\,{in}{{d}}^{-1}=({ng}\,{in}{{d}}_{0}^{-1}-{Plateau})\times {{e}}^{-{kt}}+{Plateau};$ R^2^ = 0.73 for CPs, R^2^ = 0.98 for TBBPA, Supplementary Table S5).

Different doses of CPs, TBBPA, and PFOA were delivered *to D. magna* via liposomes (Fig. 2A,B). The total concentration of liposomes in the water was the same for all treatments, which was achieved by manipulating the ratio between chemical-loaded liposomes and blank liposomes that were added to the water. Unfortunately, the body burden of PFOA in *D. magna* was under the limit of detection, and therefore no body burden or immobilization data were derived.

The body burden at the end of the 48 hour incubation increased linearly in function of the amount of TBBPA or CPs dosed via liposomes (Fig. 2A,B, R^2^ = 0.97). Mortality was observed when CPs were dosed via liposomes above 120 ng (total added to 20 mL media) and this resulted in an increase in the standard deviation of the determined body burden of CPs (ng per individual) in the animals (Supplementary Fig. S4). For this reason, a linear equation was derived, where only the sub-mortality doses were correlated to the body burden of the animals (Supplementary Table S3).

The increase of body burden over time (at a fixed amount of dosed CPs or TBBPA) was observed by taking animal samples between 4 and 48 hours of incubation with the loaded liposomes (Fig. 2C,D). When CPs were dosed via liposomes to the animals, a plateau was observed after 21 hours (Supplementary Table S4), whereas for the liposome delivered TBBPA the plateau was not reached within the 48 hours. Time to steady state for TBBPA was estimated to be reached at 91 hours of incubation (Supplementary Table S4).

After 48 hours incubation with the loaded liposomes, some animals were fed 1 mg mL^−1^ of cellulose to purge their guts of loaded liposomes, and samples were taken after 2 and 6 hours of incubation (Fig. 2E,F). By fitting the results into an exponential one phase decay model, the half-lives under these conditions were estimated to be of 6.5 and 0.89 hours for CPs and TBBPA, respectively (R^2^ = 0.73 for CPs, R^2^ = 0.98 for TBBPA, Supplementary Table S5). After 2 hours, the animals are expected to have completely purged their guts from liposomes, and have substituted the gut content with new cellulose particles. The amount of chemical left in the animals after two hours was 31 and 86% of the body burden at the start of the depuration phase (*t* = 0) for TBBPA and CPs, respectively (Supplementary Table S6). Over the subsequent 4 hours the depuration occurred at similar rates for TBBPA and CPs (0.94 and 0.41 ng eliminated per hour, respectively, Supplementary Table S6).

**Figure 3:**
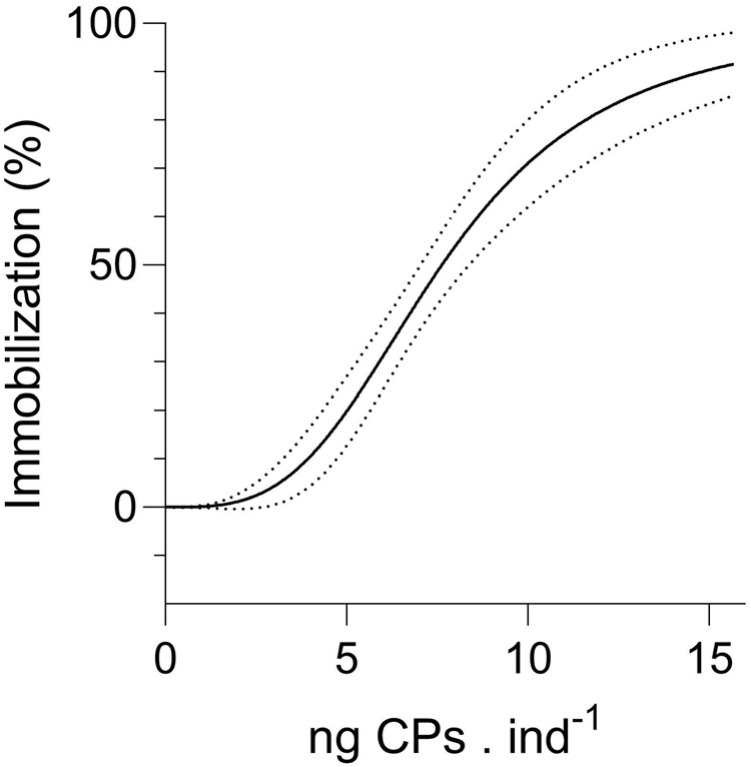
Dose-response curve for *D. magna* with increasing body burden of CPs (ng CPs per individual) dosed via liposomes (${Immobilization}=\frac{100}{1+{10}^{{\boldsymbol{logL}}{{\boldsymbol{D}}}_{50}-{\boldsymbol{ng}}{\boldsymbol{CPs}}{\boldsymbol{in}}{{\boldsymbol{d}}}^{-1}}};$. R^2^ = 0.77, Supplementary Table S7). Groups of 10 individuals (n = 3) were exposed to increasing doses of CPs via liposomes at a constant liposome concentration of 0.5 × 10^5^ liposomes mL^−1^. 95% confidence intervals of the curve are dotted.

A dose-dependent immobilization of *D. magna* was observed when the animals were incubated with increasing concentrations of CP-loaded liposomes. A lethal dose, LD_50_, of 7.6 ng CPs per individual was derived (Supplementary Table S7, Fig. 3). No dose-dependent immobilization was observed when TBBPA- and PFOA-loaded liposomes were fed to the animals. However, a dose-response curve was obtained for water-dosed TBBPA (Supplementary Fig. S5) from which a comparison could be made between the body burdens of TBBPA when dosed directly via water or via liposomes (Supplementary Fig. S6). The observed water-based EC_50_ was calculated to be at 1.6 mg L^−1^ (Supplementary Fig. S5), and the estimated body burden at EC_50_ was observed to be nearly 150 ng TBBPA per animal (Supplementary Fig. S6). The maximum observed body burden for liposome-delivered TBBPA was 20 ng per individual.

## Discussion

CPs proved highly suitable for incorporation in liposomes with a high incorporation potential, high recoveries, and even a slightly higher production yield of liposomes compared to the control. The ease at which CPs can be encapsulated in liposomes is likely dependent on the high log *K*_*ow*_ and relatively small size, which means that they can be trapped in the hydrophobic intermembrane space^14,23^. On the other hand, TBBPA, as an ionic chemical, is expected to be mostly in its anionic form (pH of M7 medium varies between 7.4 and 7.8) and encapsulated in the aqueous vacuole of the liposome^14,22^. However, the high encapsulation yield for the TBBPA (84%) suggest a high affinity for the liposomes. Considering that the liposomes were prepared using 130 mg of lipids in 4 mL of water, an 84% recovery in the liposomes would mean a liposome/water partition coefficient (*K*_*lip/w*_) for TBBPA of around 160, which means that the incorporation cannot be solely explained by encapsulation in the aqueous vacuole.

PFOA, as an amphipathic chemical, is expected to be trapped together with the phospholipids in the liposome bilayer^24^. PFOA liposomes showed the lowest production yield, incorporation, and stability over time. This is likely a result of the surfactant characteristic of PFOA^25^, not only preventing the formation of liposomes, but also destabilizing the lipid bilayer leading to their disintegration over time. Considering that only 30% of the PFOA was incorporated into the liposomes at 5 mg g^−1^, the maximum amount of PFOA possible to incorporate into the liposomes without significantly compromising their stability is, in fact, around 1.5 mg g^−1^. At this concentration, PFOA-loaded liposomes are relatively more stable, however, considerably high leakage was observed when they were diluted to test concentrations in M7 media.

Leakage of the loaded chemicals from the liposomes to the M7 medium was observed CP and TBBPA as well, but at a much smaller magnitude. Our results indicate that chemiosmosis following the initial dilution is the major factor driving this leakage, leading to an immediate release of a part of the loaded chemical. The stabilization of the leakage following the dilution and over the following 120 hours suggests that equilibration occurs rapidly. However, it is also possible that the chemicals are continuously leaking from the liposomes over the course of the 120 hours and partitioning to the borosilicate walls of the vial. Nonetheless, the observed leakage means that exposure to chemicals is not only via liposomes but also, to a smaller extent, via water.

Our observations suggest that the increased immobilization of the daphnids exposed to blank liposome concentrations ≥1 × 10^5^ particles mL^−1^ was caused by strong electrostatic interactions between the liposomes and the chitin exoskeleton of the daphnids, and/or by aggregation of liposomes in their setulae or antenna. This aggregation, which is concentration-dependent^26^, ultimately hinders the animal’s swimming ability and therefore immobilization is observed. The liposome concentration in water used in this study was optimized to guarantee no significant decreases in survival during the 48 hour incubation. A liposome concentration of 0.5 × 10^5^ liposomes mL^−1^ is nearly 10-fold lower than the concentration used by Buttino and co-workers for copepods, however, they used an incubation period of only 24 hours instead of 48^22^. In this study no differences in ingestion rate (a well-established sensitive sub-lethal endpoint^27^) between algae and blank liposomes were observed. This corroborates that the increased mortality observed after exposure to high concentrations of blank liposomes is likely a result of particle aggregation in vital swimming apparatuses. It was expected that daphnids would ingest liposomes considering that, as passive filter feeders, daphnids’ ingestion rate of particles in suspension is mostly dependent on concentration^28^ and size^29^, and studies indicate that there is no particle preference^26,30^. On the other hand, decreases in feeding/particle ingestion as a result of chemical exposure are well-described^27,28,31^. The ingestion rate was significantly lower for TBBPA and PFOA loaded liposomes. We cannot confirm whether this is a toxicological response or a particle/food preference. Moreover, *D. magna* are capable of filter feeding on particles as small as 0.2 μm and as large as 50 µm, with the highest efficiency between 0.7 and 40 μm, based on the distances between setulae, which are used as filters^29^. Additionally, in *D. magna*, the mesh-size of these filters does not increase significantly with growth (age), in contrast to other Cladocera^29^. *D. magna* can feed on bacterial food (<2 μm) as efficiently as on unicellular algae (2–50 μm)^20,32^, which means that size range of the liposomes produced in this study is appropriate for testing with *D. magna*.

A dose-dependent increase in body burden was observed for TBBPA and CPs, but not for PFOA as it was under the limit of detection. Steady-state body burden was achieved within the 48 hour incubation for CPs but not for TBBPA, while depuration was much faster for TBBPA. This suggests that the uptake of CPs from the liposomes might be different from the uptake of TBBPA. CPs are likely taken up by both equilibrium partitioning to the gut of the animals and, later, by digestion of the liposomes and release of the chemicals into their gut. On the other hand, for TBBPA, the equilibrium partitioning might be limited due to the intrinsic chemical properties of the chemical (i.e., higher water solubility, lower *K*_ow_) and therefore uptake may have to involve digestion of the liposomes. Cellulose was chosen as a particle to depurate the guts of the animals since it offers the advantage of being an organic particle that does not contribute to growth or metabolism-driven elimination (as it is a non-lipidic complex polysaccharide, as opposed to algae cells)^33,34^. It was also previously observed *D. magna*’s gut can be cleared of non-natural particles (>90%) in just under 12 minutes^26^. During the depuration with cellulose particles, it is expected that the gut’s liposome content will be substituted by new particles eliminating undigested liposomes. Thus, the decrease in body burden after two hours is expected to be a result of i) The elimination of loaded liposomes from their gut (which were contributing to the body burden) and ii) Chemical elimination by the animal (via e.g., metabolism or feces). Whereas the elimination between 2 and 6 hours, the is expected to be driven solely by (ii), i.e. metabolism and/or excretion via feces.

The observed concentration-dependent immobilization for the liposome-delivered CPs (toxicological response, Fig. 3) confirms that the CPs are being taken up by the animals. It has previously been demonstrated that crustaceans are able to digest and take up liposome-delivered fatty acids, by confirming and following the elongation of deuterated C18:3ω3 over 24, 48 and 96 hours of incubation up to C22:6ω3^21^. The LD_50_ determined for liposome-delivered CPs was 7.6 ng per individual, which corresponds to approximately 1 ng CPs μg dw^−1^. In comparison, in *D. magna* exposed to the same CP substance as used in this study (CP-52) but freely dissolved in water via passive dosing, less than 10% immobilization was observed up to 10 ± 1.5 ng μg dw^−1^ ^9^. Thus, liposome-mediated delivery of CPs caused immobilization at a much lower body burden than passive dosed-CPs via water. Such differences might be attributed to: i) Steady-state body burdens being more quickly established via liposome delivered-CPs (21 *vs* 40 hours^34^ for liposome-delivered CPs and passive-dosed CPs respectively), ii) Direct uptake to specific, and possibly, critical tissues of the animal (i.e., tissues surrounding the gut or the gut itself), and/or iii) Mixture toxicity effects from CPs plus the energy-costing clearance of particles with no nutritious value. Passive dosing is a well-established testing method for hydrophobic chemicals, including CPs, with a quasi-inexhaustible source of chemicals (silicone) that allows the establishment of complex multi-matrix equilibriums. While, in a passive dosing system, the free concentration of a given chemical in water can be directly translated to the free concentration in water in an ecosystem (which is more often used during environmental risk assessment), toxicity estimates from liposome delivery needs careful interpretation as it mostly mimics exposure via food.

In this study, no concentration-dependent immobilization was observed for liposome delivered TBBPA and PFOA, although a lowered ingestion rate of TBBPA and PFOA-loaded liposomes was observed. To achieve a toxicological response in immobilization for TBBPA and PFOA, higher exposure doses of these chemicals would be needed. However, the use of higher doses is hindered both by the maximum concentration of chemicals that can be incorporated in the liposomes, without destabilizing the liposomes, as well as by the maximum concentration of liposomes that can be added to the water, without significantly increasing immobilization in the control. Although the ingestion of liposomes could limit the ability to achieve body burdens that exert effects in apical endpoints (e.g., survival), this approach could still be useful to detect changes in other subcellular endpoints of toxicological interest. In this study the exposure concentrations were high enough to detect effects in ingestion rate even for TBBPA and PFOA.

While this study describes yet another method that could be used to address the effects of hydrophobic chemicals in aquatic biota (possibly, as a surrogate for food), the development of alternative and reliable aquatic toxicity testing methods for ionic and amphipathic chemicals such as TBBPA and PFOA, respectively, is still badly needed. The availability in water of ionic chemicals varies greatly with pH, which ultimately adds uncertainties when determining toxicity estimates for the environmental hazard assessment of these chemicals. As an additional advantage, liposome−water distribution of ionic compounds are much less dependent on pH^35^. For PFOA, the current acute toxicity estimates determined for *D. magna*^36,37^ are in the same range as low-toxicity narcotic chemicals such as alcohols^38^. These toxicity estimates are likely underestimated, considering the well-known toxicity potential of PFOA^39^. Moreover, perfluoroalkyl substances are well-known for their challenging properties and are currently a huge class of chemicals including more than 4000 substances^40^.

Even though the incorporation and stabilization of PFOA (or similar chemicals) in the liposomes needs to be optimized in the future, this study shows that it is possible to use this approach to deliver chemicals to one of the most widely used test organisms in aquatic toxicology and hazard assessment of chemicals (*D. magna*). If improved, liposomes could offer a versatile alternative to understand metabolism and fate of these types of chemicals in aquatic biota. Other sources of lipids for the liposome bilayer could be tested to improve incorporation and stability of amphiphile loaded liposomes. In this study only lecithin from soybean and cholesterol were used to create the artificial lipidic bilayer. This was in order to produce cost effective liposomes with low toxicity that could be used in routine toxicity tests. However, the lecithin is quite generic and other lipid sources with specific polar groups, chain lengths, and saturation levels are available that could probably be used to optimize incorporation of specific chemicals.

## Materials and methods

### Chemicals and materials

The immobilization experiments were conducted in 24 mL scintillation vials from Wheaton (VWR, Radnor, PA, EUA). Nile Red and analytical grade dichloromethane, acetone and methanol were purchased from Merck Millipore Laboratories (Merck KGaA, Darmstadt, Germany). Milli Q water was obtained using a Q-POD Ultrapure Water Remote Dispenser (Merck KGaA). M7 medium was prepared according to OECD standards 202^41^ and 211^42^. Hionic-Fluor and Soluene were purchased from Perkin Elmer (Waltham, MA, USA). Stainless steel surgical scalpel blades (nr. 10) were purchased from Swann-Morton (Sheffield, United Kingdom). Cellulose powder (2–20 μm) was purchased from Macherey-Nagel GmbH & Co. KG (Düren, Germany). Lecithin from soybean (90%) was acquired from PanReac AppliChem (Darmstadt, Germany). Cholesterol (99.5%) was purchased from Eastman Chemical Company (Kingsport, TN, USA). TBBPA (97%) was purchased from Sigma-Aldrich (Saint Louis, MO, USA), ^14^C-labelled TBBPA was synthesized by Halldin and co-workers^43^. PFOA (97%) was also acquired from Sigma-Aldrich, and was converted to its sodium salt by reaction with sodium hydrogen carbonate (99.7%, Sigma-Aldrich) in methanol. ^14^C-labelled PFOA was synthesized in-house^44^. The technical chlorinated paraffin mix used in this study, CP-52 (C_9_–_30_, average 52% chlorine content (w/w)), was obtained from China^34^. ^14^C-labelled perchlorododecane was synthesized Bergman and co-workers^45^. Non-labelled and ^14^C- chemicals were mixed to a specific activity of 11.35 µCi/mg for TBBPA, 15.93 µCi/mg for PFOA and 1.61 µCi/mg for CPs.

### Radiometric analysis

To quantify the ^14^C-labelled chemicals, radiometric measurements were conducted on a Tri-Carb 2100TR liquid scintillation analyzer from Packard (Perkin Elmer, Waltham, MA, USA) using Hionic-Fluor scintillation cocktail. Although the instrument was calibrated using an external ^14^C reference solution, external calibration curves of the ^14^C-labelled TBBPA, PFOA, and CPs were still prepared and used for quantification to ensure a linear response. The measured decay in each sample was converted into amount of chemical (TBBPA, PFOA or CPs) using the specific activity for each chemical.

### Liposome preparation

The liposomes in this work were prepared as published elsewhere^22^, with small modifications. Briefly, 100 mg of lecithin and 30 mg of cholesterol were dissolved in a mixture of dichloromethane (2.7 mL) and acetone (1.3 mL) in a 250 mL round bottomed flask. To produce the chemical-loaded liposomes the test substance was added to this mixture. The chlorinated paraffins were added dissolved in dichloromethane whereas the PFOA (as its sodium salt) and TBBPA were added dissolved in methanol. The solvents were then removed under a stream of nitrogen while rotating the flask to achieve a thin lipid film covering the inside of the flask. Liposomes were formed by removing the homogenous lipid film of lecithin, cholesterol and the test substance from the walls of the flask into M7 medium (4 mL), with the aid of 8 glass beads (2 mm in diameter). The flask was then rotated for 30 min and left to rest between 2 and 3 hours according to Buttino and co-workers^22^. The liposome slurry was then divided in two and transferred to two 2 mL Eppendorf tubes before they were centrifuged at 7200 *g* for 20 min. The M7 medium was then discarded and liposomes were resuspended in an equal amount of fresh M7. This procedure was repeated once more to clean the liposomes before finally resuspending them in a total 13 mL of M7 medium in a glass test tube. Liposomes were kept in glass tubes and protected from light at 5 °C until use.

### Liposome characterization

Three unlabeled, chemical-free batches of liposomes were prepared (blank liposomes). Liposome concentration was determined using an automated cell counter (TC20 Cell Counter, Bio-Rad, Hercules, CA, USA). Liposome size distribution was measured with a Spectrex laser particle counter (Spectrex, model PC-2000, Redwood City, USA), using in-built filter F0 (1–100 µm). The raw size distribution spectra obtained from Spectrex was analyzed in GRADISTAT developped by Blott and Pye^46^. All particle size distribution metrics can be found in Supplementary Table S1. The size distribution of the liposomes followed a unimodal distribution, however, as the size distribution was not normally distributed, the liposomes are described by median and 10^th^ and 90^th^ percentile ranges in Supplementary Table S1. Concentration of the chemical-loaded liposomes was measured right after production and one and three months after preparation, in order to study the stability of the synthesized liposomes (see Fig. 1 and Supplementary Table S1).

### Encapsulation efficiency

In order to evaluate encapsulation efficiency of the chemicals into the liposomes an aliquot sample of the prepared liposomes was taken to determine the incorporated radioactivity. The activity in the liposomes was then compared to the activity of a reference sample, created from the pure standard, to determine the yield. All M7 water used to create and wash the liposomes was also collected and measured for radioactivity.

### Animal maintenance and collection

*D. magna* was used as test organism (originally from the Federal Environment Agency, Berlin, Germany). *D. magna* neonates used in this experiment were obtained from a running culture of *D. magna*. The running culture was kept in synthetic freshwater (M7 culture medium, OECD 211) with a density of approximately 10 individuals L^−1^ and fed three times per week with *Pseudokirchneriella subcapitata*. The algae were cultured in MBL culture medium (OECD 201) on a shaking table with constant light (70 μE cm^−2^ s ^−1^) and temperature (18 °C).

### Body burden and immobilization experiments

**Figure 4:**
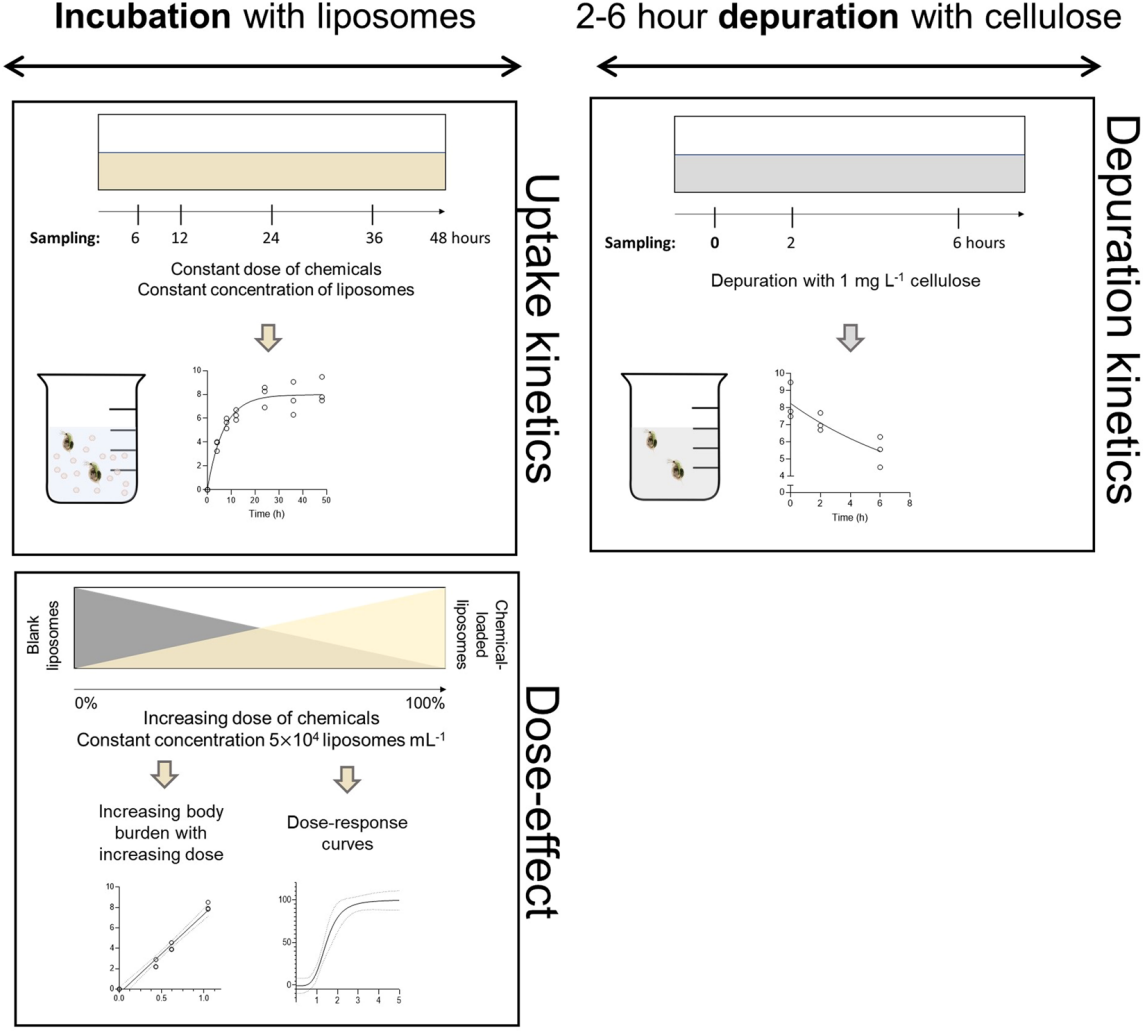
Illustration of the experimental design of the body burden and immobilization experiments. The uptake (exposure) phase lasted 48 hours and was followed by the depuration (post-exposure, 2 to 6 hours) phase. Uptake kinetics of liposome-mediated delivery of chemicals to *D. magna* were determined by sampling *D. magna* between 4 to 48 hours of incubation (left panel). Dose-effect (i.e., dose-body burden and dose-response) relationships where obtained by using increasing ratios of chemical loaded liposomes (% of total liposomes, bottom panel). Depuration kinetics were determined by moving exposed daphnids to liposome-free media and allowed to depurate with cellulose (right panel).

The toxicity and internal dose assessment experiments were based on the OECD test number 202 (*Daphnia* sp. Acute Immobilization Test). Daphnid neonates (aged < 24 h) were collected and transferred to the 24 mL vials with M7 culture medium and blank and/or chemical loaded liposomes in groups of 10 individuals (2 mL medium per daphnid). Three technical replicates were used for all treatments. The liposome concentration during the experiments was kept constant at 0.5 × 10^5^ liposomes mL^−1^, by manipulating the ratio between blank and chemical-loaded liposomes (TBBPA, CPs, PFOA (Fig. 4)). This is important since the particle ingestion rate of daphnids is largely determined by particle concentration^28^. The optimal liposome concentration for the body burden and immobilization experiments was determined prior to the ecotoxicity tests, during which several liposome concentrations were tested for 48 hours, from 5 × 10^5^–0.5 × 10^5^ liposomes mL^−1^ (Supplementary Fig. S1). The concentration used for food particles during culture maintenance was 2.5 × 10^5^ particles mL^−1^.

The acute immobilization tests were carried out under static conditions, and the culture conditions (light, medium, temperature) were similar to those as used for the stock culture. The animals were kept at a constant temperature of 22 °C throughout the experiment, with a 16:8 h light/dark cycle (light intensity of 20 μE m^−2^ s^−1^). Immobilization was recorded at 48 hours, by counting the animals that were unable to swim within 15 seconds after gentle agitation of the test vials, according to OECD 202 guideline (*Daphnia* sp. Acute Immobilization Test). At the end of the 48 hour test, the live and (if present) dead animals were collected after exposure to the chemical-loaded liposomes (^14^C-labelled chemicals) for the body burden analysis.

To observe uptake kinetics of the chemicals in the animals, samples were taken between 4 and 48 hours incubation at constant liposome and chemical concentration (Fig. 4, left panel). The data was fitted with an exponential plateau model and the output parameters were used to calculate C_steady-state_ (95% Y_M_) and the time to C_steady-state_ (*h*) with the model’s equation (Supplementary Table S4). Additionally, another experiment was performed to evaluate depuration kinetics and how much of the chemical is internalized by the animals after 48 hours of incubation (Fig. 4, right panel). For that, the animals’ gut was purged by moving them to M7 medium with 1 mg L^−1^ cellulose and samples were collected after 2 and 6 hours incubation. After 2 hours incubation, the entire gut content is completely substituted by cellulose, eliminating the undigested liposomes (with ^14^C-labelled chemicals) that are contributing to body burden. The gut evacuation under two hours was confirmed separately by looking at the daphnids under the microscope (Leica DMR Fluorescent Microscope (Leica Camera AG, Wetzlar, Germany). Pictures were taken using a Nikon D50 (Supplementary Fig. S7), showing the substitution of algae cells (green, left animal in Supplementary Fig. S7) by cellulose particles (white, right animal in Supplementary Fig. S7). The data were fitted in an exponential one-phase decay model (GraphPad) and the output parameters can be found in Supplementary Table S5. Half-life was calculated directly from the model and given as a model output (Supplementary Table S5).

### Body burden analysis

For the body burden analysis (ng chemical per individual *D. magna*), the collected animals were gently washed with clean M7 medium in order to remove any liposomes attached to the exoskeleton. The animals were then collected into a clean scintillation vial, mashed with a stainless-steel surgical blade, and left to dissolve overnight in Soluene (1 mL). Before analysis, Hionic-Fluor (10 mL) was added. One blank sample was prepared per experiment (animals exposed only to blank liposomes (no ^14^C-labelled chemicals). All samples were corrected for the blank values.

### Leakage experiment

In order to evaluate leaking of the organic chemicals from the liposomes, the chemical-loaded liposome slurry (10 mg liposomes mL^−1^ M7) was added to fresh M7 medium at a ratio of 1:3000, resulting in a concentration of 0.5 × 10^5^ liposomes mL^−1^. 1 mL samples (liposomes + M7 medium) were collected after 0, 24, 48 and 120 hours of incubation. The samples were centrifuged at 7200 *g* and the water was further filtered through a 0.45 µm syringe filter before the radioactivity was measure. The leakage was determined by comparing the radioactivity in the water at each time point with the radioactivity in 1 mL non-filtered water (i.e. water plus liposomes) at time 0.

### Liposome ingestion experiment

Liposome ingestion experiments were performed to evaluate feeding preference of *D. magna* between algae and liposomes, based on the test developed by Barata and co-workers^27^. To evaluate differences in particle ingestion rate, algae and chemical-free and chemical-loaded liposomes (TBBPA, CPs, PFOA) were added separately in 24 mL scintillation glass vials to make up a similar initial concentration of 1 × 10^5^ particle mL^−1^. Four technical replicates per treatment were used. Five *D. magna* juveniles (4 to 5 days old) were collected from the stock culture and added to each glass vial. One vial per treatment with no animals, only algae or liposomes in M7 medium, was included to account for losses due to adsorption and/or decomposition of the organic particles. After 8 hours, resulting concentrations in water of algae and liposomes were measured with a cell counter (TC20 Cell Counter).

Confirmation of ingestion of liposome particles was done separately. The liposomes were stained with Nile Red (NR) at 1.5 μM from a NR stock solution dissolved in DMSO. The liposomes were added to the M7 media at the same concentration (1 × 10^5^ particle mL^−1^), and thereafter daphnid juveniles were added. After 18 hours, NR fluorescence was observed in each individual separately using a Leica DMR Fluorescence microscope with a BG38 filter and built-in fluorescent filters. Pictures were taken using a NikonD50 (Supplementary Fig. S3). Fluorescence in the gut indicates ingestion of the stained liposomes.

### Statistical analysis

GraphPad Prism version 8.3.0 (GraphPad Software, La Jolla, California, USA) was used for all the statistical analysis and plotting in this study, with the level of statistical significance set to p < 0.05. A simple linear regression was used to visualize the body burden as a function of the amount of chemical (μg) added to the system via liposomes after 48 hours incubation. To calculate time to steady state and time to half-life (depuration) of the chemicals in the animals, data was fitted in an exponential plateau and exponential one-phase decay models, respectively, with no special handling of outliers and with the only constraint that rate constants are bigger than 0 (*k* > 0). Lethal dose (LD_50_) and the corresponding 95% confidence intervals were calculated by fitting immobilization data in a four-parameter dose-response curve with set constraints for top (=100% immobilization) and bottom (=0% immobilization) values. To compare differences between ingestion rates in the animals exposed to algae, blank and chemical loaded liposomes, a non-parametric Kruskal-Wallis test was performed followed by Dunn’s multiple comparisons test, with blank liposomes set as a control.

## Supplementary information

Supplementary information.
