# Supplementary material for: Liposome-mediated delivery of challenging chemicals to aid environmental assessment of Bioaccumulative (B) and Toxic (T) properties
Source: Sci Rep. 2020 Jun 16;10:9725. doi: 10.1038/s41598-020-66694-3 (PMC7297709; doi:10.1038/s41598-020-66694-3)
Supplement: Supplementary file 1 — Supplementary information. [file 41598_2020_66694_MOESM1_ESM.pdf]

Supporting Information

Liposome-mediated delivery of challenging chemicals to aid environmental assessment of Bioaccumulative (B) and Toxic (T) properties

Mafalda Castro, Dennis Lindqvist*

Department of Environmental Science, Stockholm University, 106-91 Stockholm, Sweden

*dennis.lindqvist@aces.su.se

7 tables

7 figures

Table S1 – Physical characteristics of the prepared liposomes in this study. Different parameters of liposome size were calculated by plotting the raw spectra in GRADISTAT[1]: mean diameter (geometric mean, µm), sorting (standard deviation of the mean diameter), skewness, which represents the symmetry or preferential spread to one side of the average, and kurtosis, which indicates the degree of concentration of the grains relative to the average. For kurtosis, values higher than 3.7 indicate a leptokurtic (strongly peaked) distribution, smaller values a platykurtic (relatively flat) distribution.

Liposome	**Blank**	**CPs**		**PFOA**			**TBBPA**	
mg g^{-1}	**0**	**50**	**100**	**5**	**10**	**100**	**4**	**50**
10th percentile (µm)	1.3	1.2	1.1	1.2	4.5	4.6	1.2	1.2
Median (µm)	2.6	2.1	2.0	2.1	5.2	5.4	2.0	2.2
90th percentile (µm)	5.0	3.3	3.5	3.6	6.1	6.1	3.6	3.9
Skewness	0.3	0.9	0.9	0.8	1.4	0.9	0.7	0.5
Kurtosis	2.2	4.5	3.3	3.2	9.0	9.5	2.6	2.6
Yield (number of liposomes)	2.3×10^9	2.7×10^9	2.9×10^9	2.1×10^9	0.6×10^9	0.3×10^9	2.2×10^9	2.1×10^9
Yield (% of blank liposomes)	100	117	126	91	25	14	94	89
Yield after one month (number of liposomes)	2.3×10^9	2.5×10^9	2.8×10^9	2.1×10^9	0.48×10^9	0.26×10^9	1.8×10^9	1.9×10^9
Yield after three months (number of liposomes)	2.02×10^9	1.78×10^9	2.2×10^9	2.0×10^9	0.036×10^9	0.031×10^9	1.6×10^9	1.6×10^9

[1] http://www.kpal.co.uk/gradistat.html

Table S2 – Incorporation of the chemicals into the liposomes (%, amount measured in the liposomes divided by the amount added to the liposome mixture) and percentage of chemicals leaking from the liposomes into the water (%, amount of chemical in water divided by the amount of chemical in water and liposomes). The test performed with the chemical-loaded liposomes in water at 0.5x10^5 liposomes mL^{-1} and samples were taken right after the liposomes were added to the water ($t_{\approx 0}$) and thereafter after one, two and five days ($t \approx$ 24, 48 and 120 hours, respectively).

Chemical loaded	mg chemical g^{-1} liposomes	Incorporation yield (%)	Leakage at $t_{\approx 0}$ (%)	Leakage at $t_{\approx 24h}$(%)	Leakage at $t_{\approx 48h}$	Leakage at $t_{\approx 120h}$
CPs	50	90	NA	NA	NA	NA
CPs	75	81	9.8	9.7	10.2	12.5
PFOA	5	30	52.7	58.1	53.5	56.2
TBBPA	50	84	22.0	25.8	27.5	25.3

NA = Not analyzed

Table S3 – Results from linear regression for the body burden (ng chemical per individual) in function of the amount of chemical (µg) added to the system via liposomes after 48 hours incubation. p-value indicates slope significantly different from zero.

	**CPs**	**TBBPA**
**Best-fit values**		
Slope	7.766	7.576
Y-intercept	-0.4087	0.3667
X-intercept	0.05263	-0.04840
**Std. Error**		
Slope	0.4048	0.3820
Y-intercept	0.2612	0.4039
**95% Confidence Intervals**		
Slope	6.864 to 8.668	6.750 to 8.401
Y-intercept	-0.9907 to 0.1733	-0.5058 to 1.239
X-intercept	-0.02466 to 0.1170	-0.1801 to 0.06138
**Goodness of Fit**		
R^2	0.9735	0.9680
P value	<0.0001	<0.0001
Equation	$Y = 7.766 \times X - 0.4087$	$Y = 7.576 \times X + 0.3667$

Table S4 – Exponential plateau model output parameters calculated from body burden (ng chemical per individual) over time (4-48 hours) at constant chemical and liposome concentration. Time to steady state is calculated from the model parameters using the model equation $Y = Y_M - (Y_M - Y_0) \times e^{-kt}$.

	**CPs**	**TBBPA**
**Best-fit values**		
Y_M	7.979	27.03
Y_0	0.07271	-0.23
k	0.1456	0.033
**Std. Error**		
Y_M	0.273	3.264
Y_0	0.414	0.762
k	0.020	0.008
**95% CI**		
Y_M	7.417 to 8.584	22.22 - 39.30
Y_0	-0.8071 to 0.9447	-1.85 - 1.36
k	0.1074 to 0.1957	0.0172 - 0.0493
**Goodness of fit**		
Degrees of Freedom	18	15
R^2	0.9395	0.9691
**$C_{steadystate}$ (95% Y_M)**	7.58	25.68
**Time to $C_{steadystate}$ (h)**	21	90.8

Table S5 - Exponential (one-phase) decay model output parameters calculated from body burden (ng chemical per individual) over time (4-48 hours) at constant chemical and liposome concentration. Time to steady state is calculated from the model parameters using the model equation $Y = (Y_0 - Plateau) \times e^{-kt} + Plateau.$

	CPs	TBBPA
**Best-fit values**		
Y0	8.255	21.03
Plateau	2.370	2.124
K	0.1074	0.7797
Half Life	6.456	0.8890
Tau	9.314	1.283
Span	5.885	18.91
**Std.error**		
Y0	0.495	0.825
Plateau	12.344	0.939
K	0.313	0.150
Half Life		
Tau		
Span	12.139	1.239
**Goodness of fit**		
Degrees of Freedom	6	6
R^2	0.7285	0.9796

Table S6 – Summary of the different conditions of chemical-loaded liposomes in which *D. magna* was incubated with (i.e., total number of liposomes in 20 mL, total chemical dosed via liposomes in 20 mL (μg)), measured ingestion rate of the different and ng of each chemical per individual after 48-hour incubation and after 2 and 6 hours of depuration with cellulose. These values were used to estimate the number of liposomes consumed per animal after 48hours, the % of liposomes consumed in 48h, the amount of chemical ingested via liposomes after 48 hours, as well as the amount and % of chemical depurated after 2 and 6 hours of depuration.

	**TBBPA**	**PFOA**	**CPs**
Ingestion rate	457±215	560±68	821±122
Liposomes consumed per animal after 48h	2.2×10^5	2.7×10^5	3.9×10^5
Total liposomes in 20 mL	1×10^6	1×10^6	1×10^6
% liposomes consumed in 48h	22	27	39
% liposomes consumed per animal in 48h	2.2	2.7	3.9
μg liposomes per mL during test	2.27	2.27	2.27
μg chemical per mL during test	0.0955	0.0034	0.1381
Total μg chemical dosed	1.91	0.0682	1.38
Estimated consumed ng / animal	42	1.9	54
**Measured**			
ng chemical per individual after 48h	16.9±2.2	0.041±0.008	8.3±1.1
% ng per individual / ng dosed	1.1	0.06	0.6
ng chemical after 2h depuration	6.1±0.97	NA	7.11±0.52
% ng per individual after 2h	31.3	NA	86.2
ng chemical after 6h depuration	2.3±0.48	NA	5.5±0.89
% ng per individual after 6h	11.8	NA	66.1

NA = Not analyzed

Table S7 – Results from dose-response curve ($Immobilization = \frac{100}{1+10^{logLD_{50}-ng\ CPs\ ind^{-1}}}$)for *D. magna* with increasing body burden (ng CPs per individual, Figure 3 in main text).

**Best-fit values**	
Hillslope	3.313
LD_{50}	7.63
**95% CI**	
Hillslope	2.392 to 4.56
LD_{50}	7.029 to 8.383
**Goodness of Fit**	
Degrees of Freedom	39
R^2	0.77

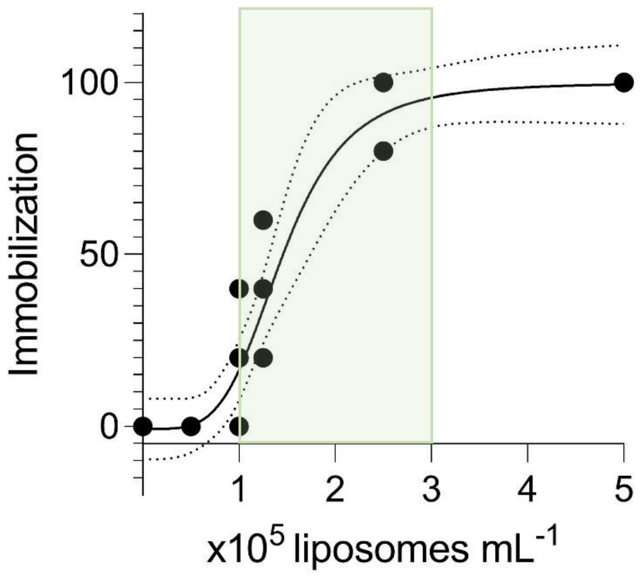

Figure S1 – Dose-response curve for the immobilization of *D. magna* with increasing liposome concentration in water (0.5 to 5 × 10^5 liposomes mL^{-1}). Groups of 10 individuals (n=4) were exposed to increasing doses of liposomes in water and immobilization was fitted in a four-parameter dose-response curve. Highlighted in green is the concentration interval for algal cells usually fed to *D. magna* in the running culture or tests. 95% confidence intervals of the curve are dotted. EC_{50} was calculated to be at 1.5 × 10^5 liposomes mL^{-1} (R^2= 0.94).

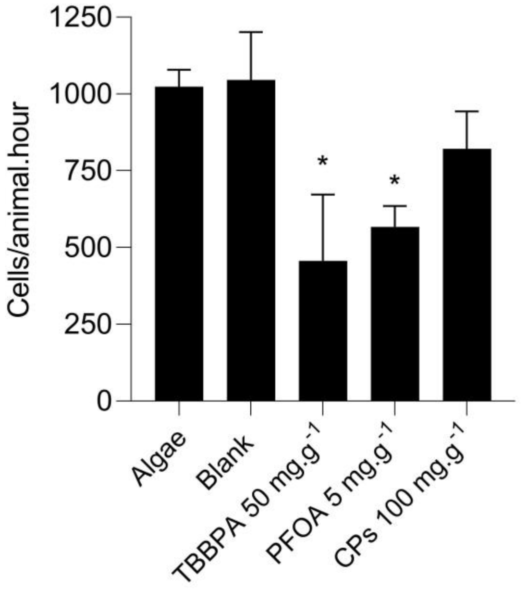

Figure S2 - Particle (liposome or food) ingestion rate in 4-5 days old *D. magna*. Asterisks indicate significant differences from control (blank liposomes, *p value < 0.05), evaluated by Dunn's multiple comparisons test.

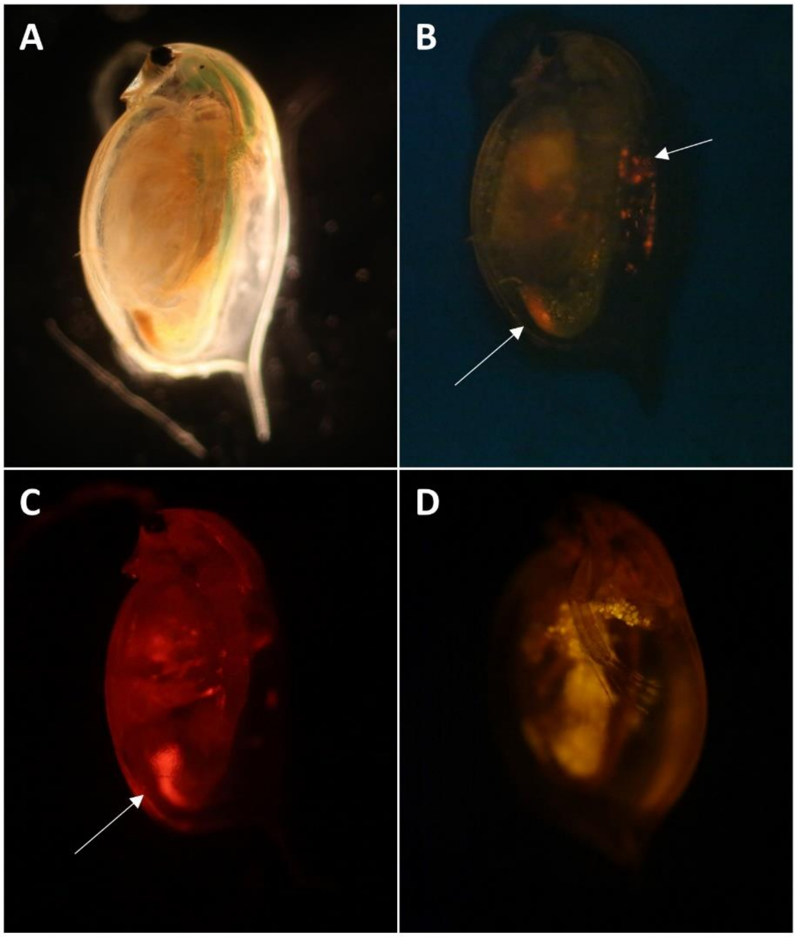

Figure S3 – *D. magna* individuals after being incubated with NR-stained liposomes for four hours. (A) *D. magna* under normal light, (B) *D. magna* incubated with NR stained liposomes (15µM) after 18 hours under blue fluorescent light, (C) *D. magna* incubated with NR stained liposomes (15µM) after 18 hours under green fluorescent light, (D) *D. magna* exposed to NR in M7 medium (15µM NR, no liposomes) after 1-hour incubation under green fluorescent light.

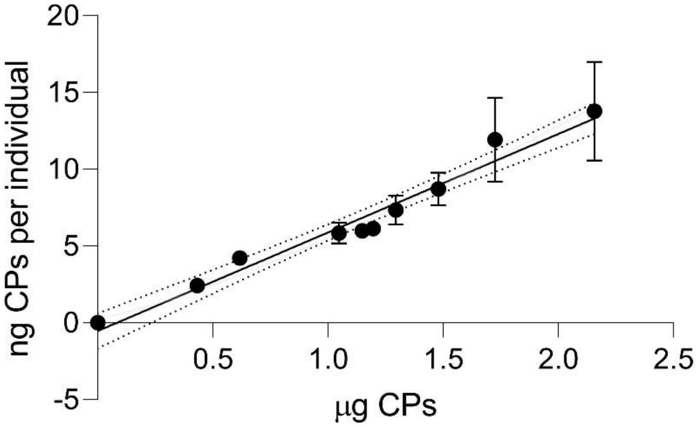

Figure S4 - Simple linear regression for the body burden in function of the amount of CPs (µg) added to the system via liposomes after 48 hours incubation (R^2=0.86) with the 95% confidence intervals of the regression dotted.

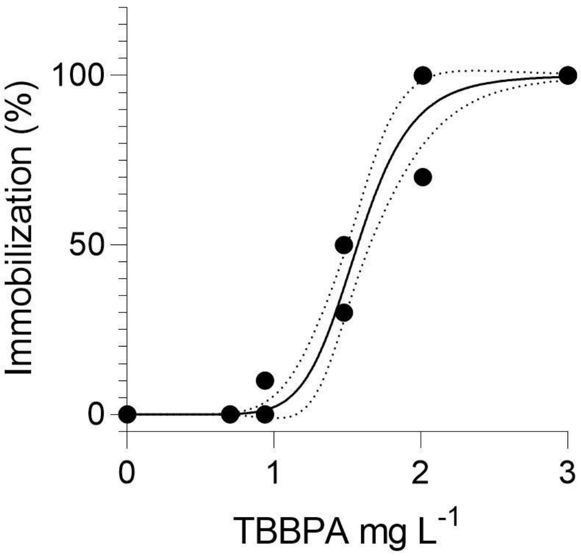

Figure S5 - Concentration-response curve for immobilization of *D. magna* with increasing water concentration of TBBPA (mg L^{-1}). Groups of 10 individuals (n=3) were exposed to increasing doses of TBBPA via water. 95% confidence intervals of the curve are dotted. EC_{50} was calculated to be at 1.570 mg L^{-1} (95% confidence intervals of the EC_{50} = 1.50 - 1.70 mg L^{-1}, Hillslope=8.4, R^2= 0.97).

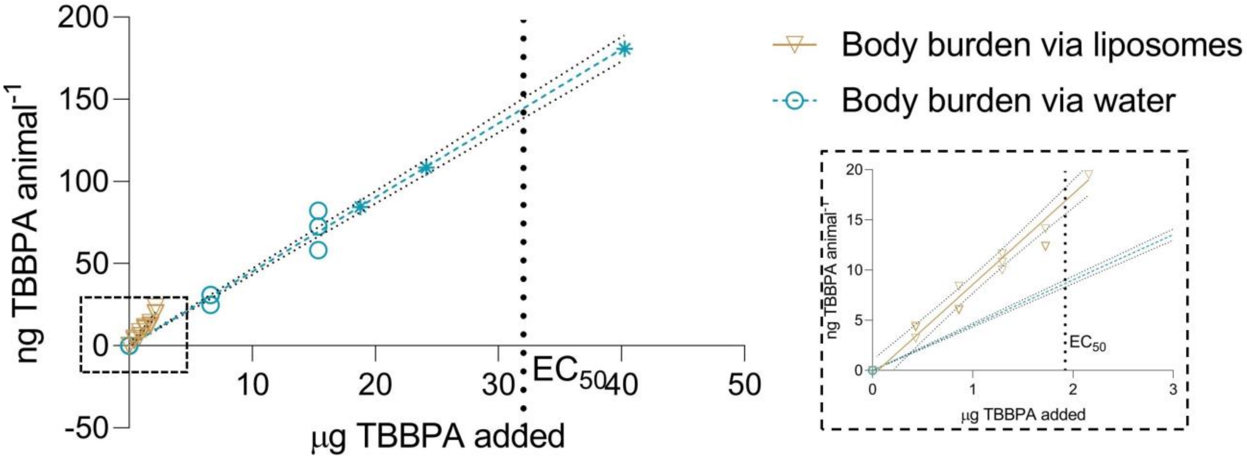

Figure S6 – Comparison of the body burden in relation to the amount of TBBPA (total μg) added via liposomes (▽) and water (○). Data points were fitted with a simple linear regression. For the body burden measured when TBBPA was delivered through water, ^{14}C-labelled TBBPA was dissolved in water and the resulting body burden was measured by analyzing the ^{14}C residues in the animal after 48-hour incubation. Due to the rapid decay of the dead animals in the water system at higher doses of TBBPA (>20 μg TBBPA), body burden measurements become quite inaccurate. Therefore, in asterisks (∗) are represented body burdens estimated from the ratio of the amount of TBBPA found in animals at lower doses divided by the total amount of TBBPA added to the system (4.49%).

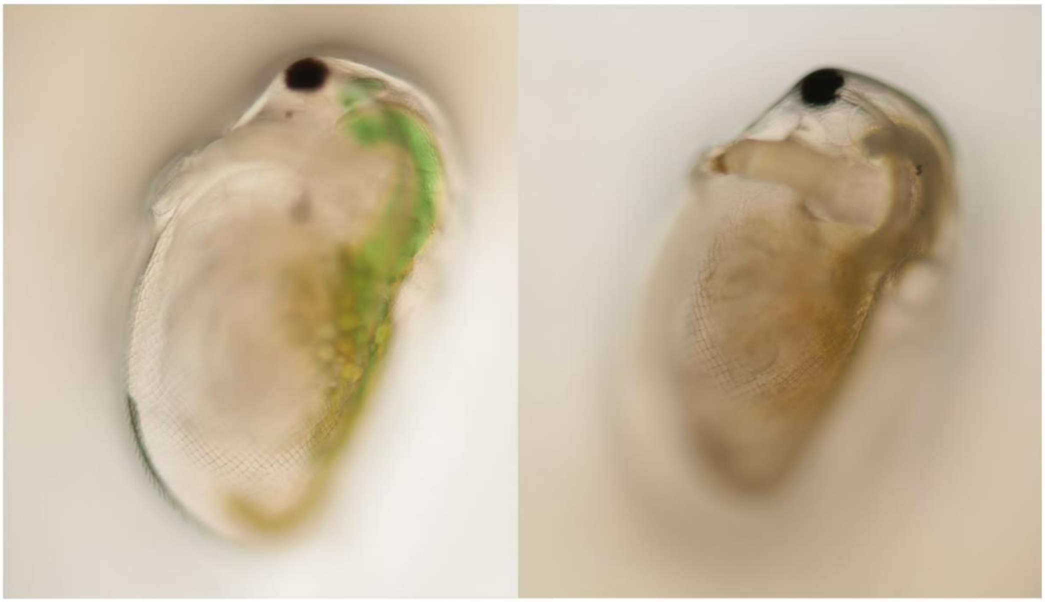

Figure S7 – *D. magna* neonate collected from the algae-fed running culture (left) and *D. magna* neonate after being fed cellulose (1 mg L^{-1}, right) for 20 hours.
